# Complications and mortality of Cushing’s disease: report on data collected over a 20-year period at a referral centre

**DOI:** 10.1007/s11102-023-01343-2

**Published:** 2023-07-26

**Authors:** Alessandro Mondin, Filippo Ceccato, Giacomo Voltan, Pierluigi Mazzeo, Renzo Manara, Luca Denaro, Carla Scaroni, Mattia Barbot

**Affiliations:** 1 Endocrinology Unit, Department of Medicine-DIMED, University Hospital of Padova, Via Ospedale Civile, 105, 35128 Padua, Italy; 2 Neuroradiology Unit, University Hospital of Padova, Padua, Italy; 3 Academic Neurosurgery, Department of Neurosciences, University of Padova, Padua, Italy

**Keywords:** Cushing, s disease, Mortality, Morbidity, Cardiovascular complications, Survival

## Abstract

**Context:**

Cushing’s disease (CD) is rare condition burdened by several systemic complications correlated to higher mortality rates. The primary goal of clinicians is to achieve remission, but it is unclear if treatment can also increase life expectancy.

**Aim:**

To assess the prevalence of cortisol-related complications and mortality in a large cohort of CD patients attending a single referral centre.

**Materials and methods:**

The clinical charts of CD patients attending a referral hospital between 2001 and 2021 were reviewed.

**Results:**

126 CD patients (median age at diagnosis 39 years) were included. At the last examination, 78/126 (61.9%) of the patients were in remission regardless of previous treatment strategies. Patients in remission showed a significant improvement in all the cardiovascular (CV) comorbidities (p < 0.05). The CV events were more frequent in older patients (p = 0.003), smokers and persistent CD groups (p < 0.05). Most of the thromboembolic (TE) and infective events occurred during active stages of the disease. The CV events were the most frequent cause of death. The standardized mortality ratio (SMR) resulted increased in persistent cases at the last follow-up (SMR 4.99, 95%CI [2.15; 9.83], p < 0.001) whilst it was not higher in those in remission (SMR 1.66, 95%CI [0.34; 4.85], p = 0.543) regardless of the timing or number of treatments carried out. A younger age at diagnosis (p = 0.005), a microadenoma (p = 0.002), and remission status at the last follow-up (p = 0.027) all increased survival. Furthermore, an elevated number of comorbidities, in particular arterial hypertension, increased mortality rates.

**Conclusions:**

Patients with active CD presented a poor survival outcome. Remission restored the patients’ life expectancy regardless of the timing or the types of treatments used to achieve it. Persistent CD-related comorbidities remained major risk factors.

**Supplementary Information:**

The online version contains supplementary material available at 10.1007/s11102-023-01343-2.

## Introduction

Cushing’s disease (CD) is the most common cause of endogenous glucocorticoid excess due to uncontrolled adrenocorticotropic hormone (ACTH) secretion from a pituitary adenoma, for the most part a microadenoma [1]. A rare condition with an estimated incidence of 0.6—2.6 cases per million per year, it is burdened by high morbidity and mortality, for the most part linked to cardiovascular (CV) events. This is particularly true for active CD which is characterized by hypertension, diabetes mellitus, obesity and dyslipidaemia. The severity of the clinical picture seems to depend more on the duration of the disease rather than on the degree of cortisol elevation, although other confounding factors may affect the clinical phenotype [2]. Prompt diagnosis and resolution of hypercortisolemia are paramount to revert cortisol-related comorbidities and to improve life expectancy. Although new individualized medical treatment options for CD continue to evolve, transsphenoidal surgery (TSS) remains the first line treatment for potentially operable patients as it is the only treatment that seems to provide a rapid, long-lasting remission. Persistent and recurrent cases are nevertheless major concerns, since up to 50% of cases might require other treatment modalities to achieve disease control and those patients are once again exposed to cortisol excess that can negatively impact their survival [3]. An increased mortality has been noted in patients with active CD, while patients in remission show a markedly lower one. It is still unclear if mortality in these patients is higher than that in the general population. Some studies report a normal life expectancy [4–8] while others describe a persistently higher mortality [9–11]. One study reported finding a higher mortality as long as 10 years after remission, and only patients cured by a single TSS showed a normal life expectancy [12].

In view of these considerations, this study was designed to assess the prevalence of cortisol-related comorbidities/complications and mortality in a large group of CD patients attending a tertiary referral centre over the past 20 years. Other study aims were to evaluate the predictors of long-term outcomes and the impact of different treatments on life expectancy in CD patients.

## Materials and Methods

One hundred twenty-six CD patients diagnosed between December 2001 and December 2021 were eligible for this monocentric, retrospective, observational study. Hypercortisolism was suspected on the basis of the patient’s clinical features and it was confirmed by appropriate hormonal testing [low dose dexamethasone suppression test (LDDST), 24-h urinary free cortisol (UFC) and late-night salivary cortisol (LNSC)] after excluding the possibility of exogenous glucocorticoid intake from any route [13]*.* UFC and LNSC were assessed at least in two different samples as recommended [14, 15]*.*

The diagnosis of ACTH-dependent syndrome was confirmed on the strength of detectable ACTH levels (> 10 ng/L) and appropriate responses to a high dose dexamethasone suppression test (HDDST), corticotrophin releasing hormone (CRH) and/or desmopressin (DDAVP) tests [16]. All the patients underwent a pituitary magnetic resonance imaging (MRI); they also underwent bilateral inferior petrosal sinus sampling (BIPSS) when the results of hormonal tests were ambiguous. The pituitary origin of ACTH secretion was confirmed by biochemical remission after TSS, histology and/or post-operative hypoadrenalism.

The results of clinical, biochemical and radiological tests as well as the treatments performed to control cortisol secretion (surgery, radiotherapy and/or medical therapy), any comorbidities (i.e., arterial hypertension, impaired glucose homeostasis, dyslipidaemia, overweight), any hormone deficiencies, any complications (i.e., CD-related events such as infective, CV and thromboembolic events) and any deaths recorded in the medical charts were collected.

The disease severity at baseline was defined on the basis of the patient’s UFC values as mild (up to two-fold the upper limit of normal – ULN), moderate (between 2 and 5 times the ULN) or severe (over five-fold the ULN).

Patient’s classification on the basis of disease activity are indicated in *Supplementary material and methods sections*.

The presence of hypertension, glucose metabolism impairment, obesity, dyslipidaemia and hypopituitarism were defined as by specific Guidelines, Supplementary [19–24].

The current study was designed in accordance with the principles of the Declaration of Helsinki and approved by the Ethical Committee of the province of Padova (protocol code 236n/AO/22, date of approval 29 April 2022).

The types of CD complications characterizing the patient were classified into three categories: CV, thromboembolic (TE), or infective (IN) events. Depending on the timing of its presentation, an event was classified as occurring: “prior” to diagnosis, “during” active CD or “after” CD remission. Events requiring hospitalization or iv antibiotic administration were registered as IN events. The causes of death were classified under the following headings: CV, infections, cancer, psychiatric complications leading to suicide, TE events or other (the last when none of the previous causes was applicable).

### Statistical analysis

Categorical variables were reported as counts or percentages, and quantitative variables as median and interquartile ranges [IQR]. The comparisons between groups were performed with a Mann–Whitney sum rank test for independent quantitative variables; a Wilcoxon signed-rank test was run for dependent quantitative variables. As far as categorical variables were concerned, the McNemar test or a chi-square test were used for paired and unpaired data, respectively.

A Cox regression analysis was performed to evaluate possible predictors for events and mortality based on the assumption of constant hazards over time. As time-dependent variables (e.g., achieving remission) did not meet this assumption, their survival analysis was performed using Kaplan–Meier analysis. Regarding complications, as there is usually a delay in CD diagnosis [25], Kaplan Meier curves for event free probability were calculated beginning 24 months prior to the diagnosis in order to include “prior” events possibly related to cortisol excess in our analysis. Vice versa, survival analysis for mortality was calculated beginning with the CD diagnosis date. Standardized mortality ratio (SMR) was calculated based on indirect age standardization in order to compare the observed deaths in our CD population with the expected number of deaths in the general population [26, 27]. A Fisher exact test was carried out to assess significant differences with respect to the general population and calculating the 95% confidence interval (95% CI) for SMR.

The threshold for statistical significance was set at p-value < 0.05. Statistical analyses were performed with R: R-4.2.0 for Windows 10 (32/64 bit) released in April 2022 and R studio desktop version 4.2.0 (2022-04-22) for Windows 10 64 bit (R Foundation for Statistical Computing, Vienna, Austria, URL https://www.R-project.org/). An open-source calculator was also used to perform the Fisher exact test (http://www.openepi.com).

## Results

### Baseline

The data of 167 CD patients attending the Centre between December 2001 and December 2021 were collected. The information regarding 41 patients were not included in the analysis because of insufficient follow-up data (i.e. patients referred for second opinion or for diagnostic workup or those with follow-up < 1 year from first line treatment). The remaining 126 patients presented a median age at diagnosis of 39 [31–50 years]; the female: male ratio was 3:1. The median follow-up was 130.5 months [72.5–201.5]. The patients’ clinical features at the time of diagnosis are outlined in Table 1.

**Table 1:** The patients’ clinical features at the time of diagnosis

Comorbidities	n/tot (%)
Arterial Hypertension	91/108 (84.3%)
Weight excess	72/96 (75.0%)
Overweight	44/96 (45.8%)
Obesity	28/96 (29.2%)
Dyslipidaemia	41/73 (56.16%)
Glucose metabolism impairment	45/87 (51.7%)
Overt diabetes	32/87 (36.8%)
Prediabetes	13/87 (14.9%)

N: the number of patients presenting the feature; tot: the number of patients whose data regarding that feature were available.

The median UFC levels were 3.2 times the ULN [2–5.6]. Almost half of the cohort presented moderate cortisol excess (45/98, 45.9%), with lower proportions of the patients presenting mild (26/98, 26.5%) and severe disease (22/98, 27.6%).

Most of the patients (91/113, 80.5%) had a microadenoma, including 29/91(31.9%) with negative imaging. The remaining 22 patients (19.5%) had a macroadenoma.

### Treatments

Most of the patients underwent TSS as the first line treatment (113/126), only one patient underwent craniotomy. Eight patients received primary medical treatment, three received first-line radiotherapy and one underwent BA soon after diagnosis. Overall, 115 patients underwent pituitary surgery (one patient with a previous unsuccessful pituitary irradiation) and the remission rate was 60.9%. Relapses were observed in 46.7% of the cases after a median time of 56 [29–83] months. The second surgery proved less successful with respect to the first one; the remission rate was 43.2% (16/37); of these, 25% developed recurrence during the follow-up period. The median time to relapse was 66.5 [36–120] months. Only two patients underwent a third surgery; in both cases it was not curative (*Supplementary Fig. 1*) [27]. A 4th and a 5th TSS were performed in one of these for debulking purposes due to an aggressive pituitary lesion. Surgical remission was not affected by pre-treatment with cortisol-lowering medications neither before the first (p = 1.0) nor the second TSS (p = 0.88). Moreover, hormone control did not improve the surgical outcomes, although a tendency towards a higher remission rate was observed in those patients who showed good disease control before undergoing the second surgery (*Supplementary Fig. 2*) [27].

Overall, 34 patients received radiotherapy, either the conventional (18.5%) or the stereotactic type (81.5%). Remission was noted in 36.7% (11/30) of the patients with at least a 12-month post-radiotherapy follow-up. As expected, the longer the follow-up, the higher the remission rate; it was 41.67% (10/24) and 46.7% (7/15) at 5 and 10 years, respectively.

Thirteen patients underwent BA and achieved complete remission. Excluding the patients with less than 12 months of follow-up, 4 out of 11 (36.4%) of the patients developed CTP-BADX/NS over a mean follow-up period of 110 [106 -329] months. Three patients out of the 11 were previously irradiated at pituitary level to control cortisol secretion. Four CD patients underwent unilateral adrenalectomy due to a dominant adrenal lesion consistent with chronic ACTH stimulation. Two (50%), harbouring unilateral adenomas larger than 5 cm, achieved remission after surgery; both cases were previously irradiated at the pituitary level.

All but one of the 48 patients with persistent hypercortisolism at the last follow-up were on cortisol lowering medications. The untreated patient had a residual mild cortisol excess after TSS and medical therapy was discontinued because of multiple drug intolerance. At the last follow-up 28 patients were receiving monotherapy, and 19 were receiving combination treatment; 25 patients were receiving steroidogenesis inhibitors, 9 pituitary-target drugs and 13 a combination of the two compounds (*Supplementary Table 1*) [27]. Most of our patients achieved UFC normalization (complete control in 67.4%, partial control in 22.7%, uncontrolled in 10.9%). Data pertaining to a single patient with renal function impairment who presented falsely low UFC were not included in this analysis. When available, LNSC was restored in 14/41 cases (34.2%). No differences in the patients’ outcomes linked to the type of treatment prescribed (monotherapy vs combination treatment) or its target (adrenal vs pituitary) were found (data not shown).

**Fig. 1:**
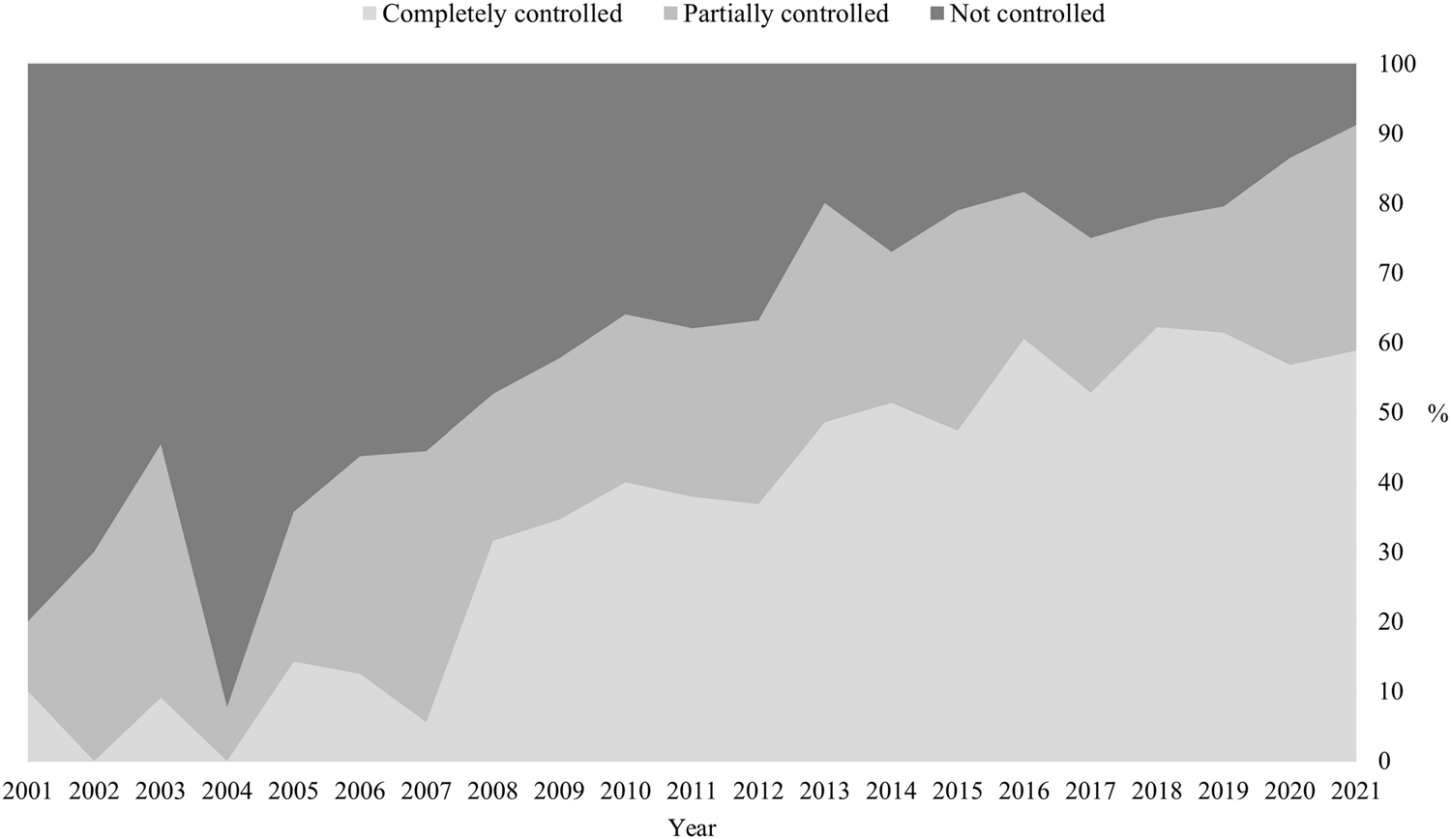
Percentage of patients controlled during active CD

We also evaluated the extent of cortisol excess throughout the active phase of CD both for the patients presenting persistence at the last available follow-up (n = 48) and for those in remission after multiple therapies (i.e., late remission) (n = 33). As described in the material and methods section, disease activity for each year of active disease was defined on the basis of patients’ UFC levels. A minimum of three UFC measurements were registered every year and the median value was calculated. When data were missing, the patients were considered uncontrolled during that period. The results are reported in *Supplementary Table 2* [27]; both the persistence and late remission groups showed UFC levels < 2xULN over more than 50% of the time span evaluated (58.8% and 73.6%, respectively). There was a progressive increase in the proportion of controlled patients over the observation period (Fig. 1).

### Comorbidities

The principal CD features at baseline and at the last follow-up examination were evaluated, (*Supplementary Table 2*). At time of diagnosis, no differences were observed as regards comorbidities between patients who achieved remission and those with persistent disease at baseline, (*Supplementary Table 3*). The patients in remission at the last examination showed a significant improvement in all the parameters considered; those with persistent CD did not (Table 2).

**Table 2:** A comparison of Cushing’s disease features at baseline and at the last follow-up examination

Feature	Disease state at the last follow-up	Baseline n/tot (%)	Last follow-up n/tot (%)	p
Hypertension	Remission	50/63 (79.4%)	20/63 (31.7%)	< 0.001*
	Persistence	38/42 (90.5%)	35/42 (83.3%)	0.371
Dyslipidaemia	Remission	23/46 (50.0%)	14/46 (30.4%)	0.039*
	Persistence	13/20 (65.0%)	14/20 (70.0%)	1
Diabetes/prediabetes	Remission	25/55 (45.5%)	15/55 (27.3%)	0.009*
	Persistence	17/28 (60.7%)	17/28 (60.7%)	1
Obesity/overweight	Remission	38/54 (70.4%)	26/54 (48.1%)	0.0015*
	Persistence	23/28 (82.1%)	22/28 (78.6%)	1

n: the number of patients presenting the condition; tot: the total number of patients presenting persistence or remission at the last follow-up examination. *p < 0.05.

As far as hormone deficiencies were concerned, 42/126 (33.3%) of the patients developed at least one deficit due to previous treatments (*Supplementary table 4*) [27], including hypocortisolism due to BA. Neither the second surgery nor radiotherapy led to an increase in hypopituitarism (*Supplementary Fig. 3*) [27].

### Complications and mortality

As far as CD complications were concerned, 18.3% of the patients had a TE event, 17.5% presented an IN event and 7.1% presented a CV one. Most of the events occurred during an active phase of CD (Table 3). Other concomitant thrombotic risk factors were present in 10/19 (52.6%) of the patients experiencing TE events. TE events were related to surgery (pituitary, adrenal or others) in 5 cases, to post-traumatic fractures in 2, to prolonged immobilization in 2, and to a symptomatic SARS CoV2 infection in one case. IN events affected the respiratory system in 9 cases, the gastro-intestinal tract in 5 cases, the soft tissues in three cases, the central nervous system in 2 cases, the musculoskeletal system in 2 cases and the genitourinary tract in one case.

**Table 3:** Thromboembolic, infective, and cardiovascular events and their timing (see materials and methods)

Type	n/tot (%)	Age
TE event		
Overall	23/126 (18.3%)	52 [36.6–63.0] years
Prior	6/23 (26.1%)	
During	12/23 (52.2%)	
After	5/23 (21.7%)	
IN event		
Overall	22/126 (17.5%)	50 [38.0–61.5] years
Prior	4/22 (18.2%)	
During	15/22 (68.2%)	
After	3/22 (13.6%)	
CV event		
Overall	9/126 (7.1%)	58 [48.5–63.0] years
Prior	2/9 (22.2%)	
During	6/9 (66.7%)	
After	1/9 (11.1%)	

n: number of patients; tot: total; TE: thromboembolic; IN: infective; CV: cardiovascular.

Overall, 11 deaths were recorded during the follow-up period (130.5 [72.5–201.5] months). The causes of death were classified as: cardiovascular events (n = 4), infections (n = 2), cancer (n = 2), suicide (n = 1), thromboembolic events (n = 0), others (n = 2; a cerebral haemorrhage in one case and an unknown cause in the other).

**Fig. 2:**
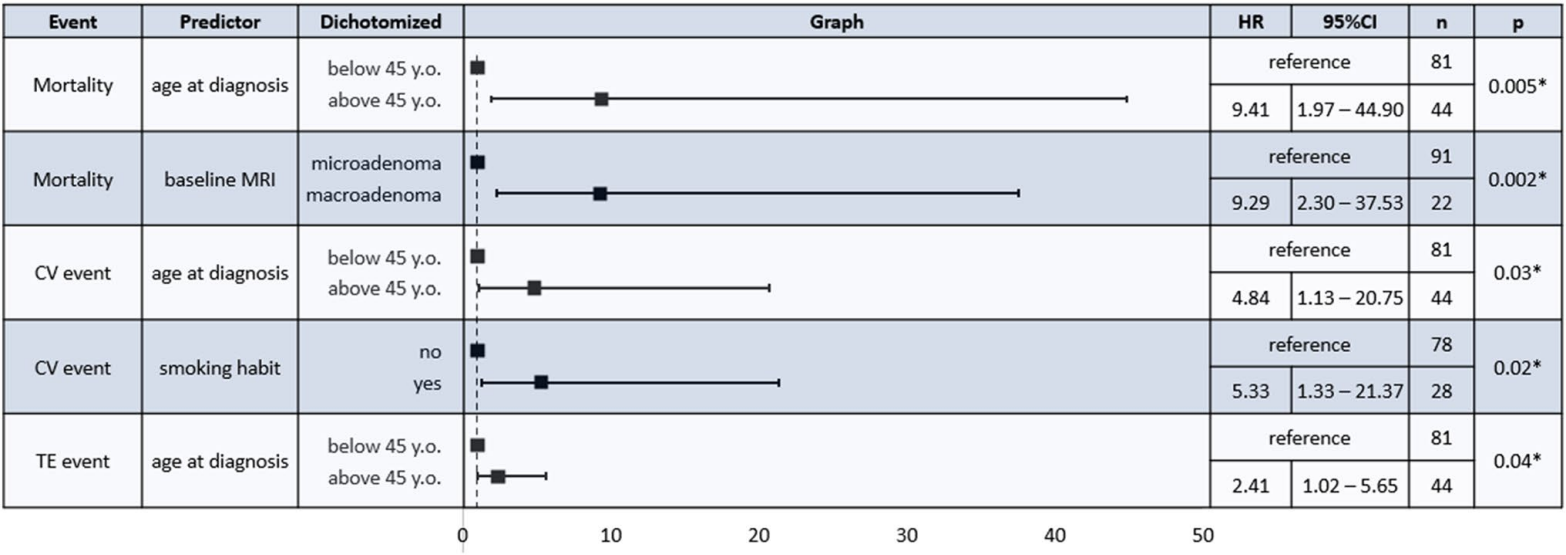
Cox regression analysis for predictors of mortality and cardiovascular, infective or thromboembolic events; only significant results are shown. HR: Hazard ratio; CI: confidence interval; n: number, CV: cardiovascular; TE: thromboembolic. *p < 0.05

Cox regression was performed to evaluate the predictors of events (CV, IN, TE) and mortality (Fig. 2). The older patients presented an increased risk of mortality (HR 9.41, 95%CI [1.97; 44.90], p = 0.005), of CV events (HR 4.84, 95%CI [1.13; 20.75], p = 0.003) and of TE events (HR 2.41, 95%CI [1.02; 5.65], p = 0.04). Similarly, the presence of a macroadenoma at the time of the first MRI was associated with reduced survival (HR 9.29, 95%CI [2.30; 37.53], p = 0.002). Smoking was correlated to CV events (HR 5.33, 95%CI [1.33; 21.37], p = 0.02). Hypercortisolism severity at baseline did not affect the risk of complications or survival. No gender related differences were observed, although a tendency toward more CV events was noted in the males (p = 0.08).

**Fig. 3:**
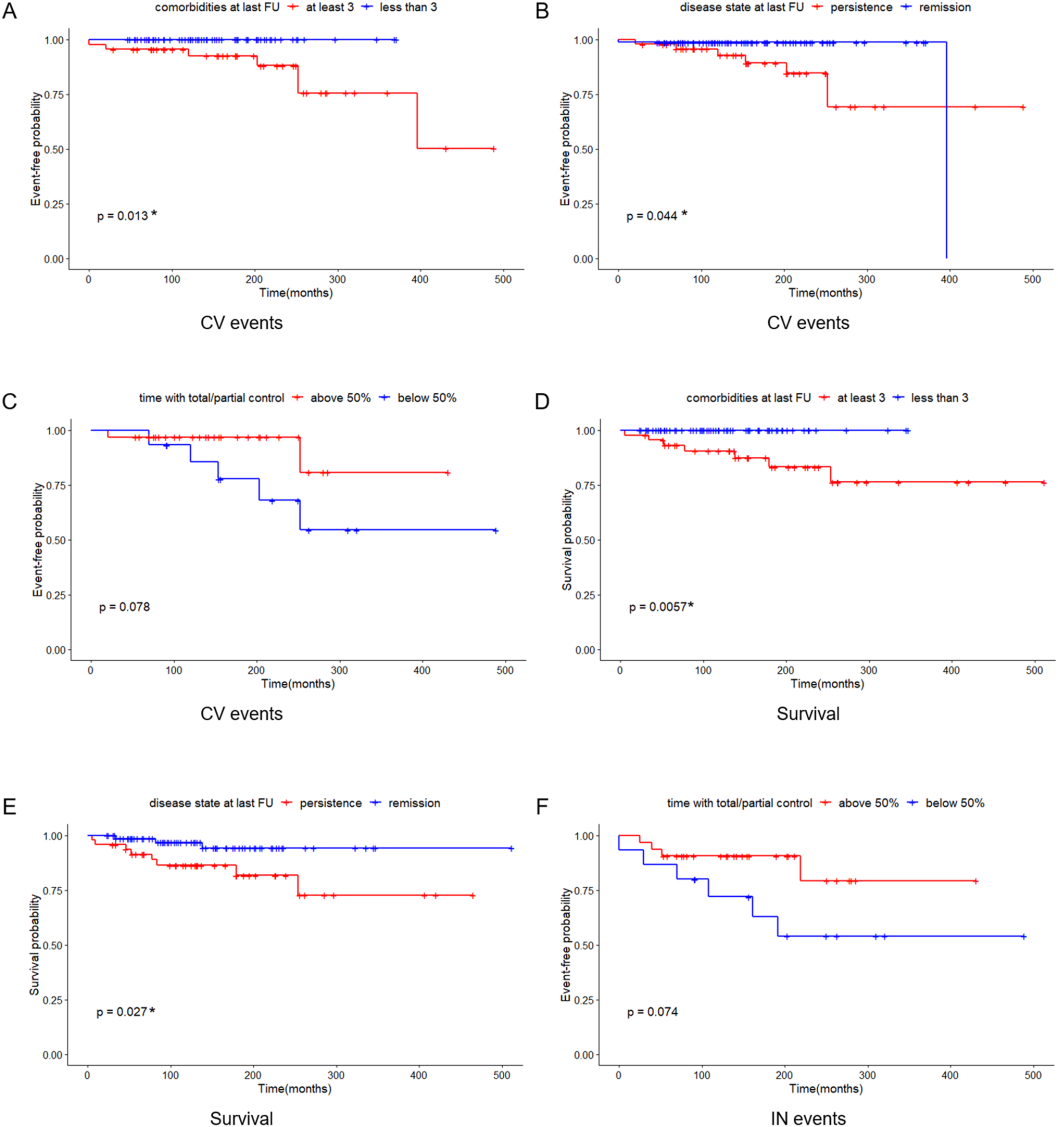
Kaplan Meier curves for cardiovascular events based on: A) comorbidities at the last follow-up examination; B) disease status at the last follow-up examination; C) control during active disease for patients presenting persistence at the last follow-up. Kaplan Meier curves for survival plotting: D) comorbidities at the last follow-up examination; E) disease status at the last follow-up examination. Kaplan Meier curves for infective events based on: F) hormone control during active disease of patients presenting persistence at the last follow-up examination. FU: follow-up; CV: cardiovascular; IN: infective. *p < 0.05

Kaplan Meier curves were plotted for complications (CV, IN and TE) and mortality in order to assess time-dependent variables (i.e., the number of comorbidities and the disease status at the last follow-up, the timing of remission and the disease activity in the patients with persistent CD at the last follow-up). We found that persistent disease and multiple comorbidities (at least 3) at the last follow-up were associated with increased CV events (p = 0.044 and p = 0.013, respectively) and mortality (p = 0.027 and p = 0.0057, respectively) (Fig. 3). The timing of remission did not influence the mortality or the risk of complications (data not shown). With regard to the patients with persistence, those presenting total/partial control for more than half of the follow-up period considered tended to have fewer CV and IN events (p = 0.078 and p = 0.074, respectively) (Fig. 3). Similarly, among patients with persistent cortisol excess the impaired circadian rhythm of secretion was associate to TE events and a trend to higher mortality (Supplementary Fig. 4). Sub-analysis of each comorbidity revealed that hypertension played a pivotal role during the follow-up period for CV complications (p = 0.011) and mortality (p = 0.0039). Similarly, dyslipidaemia was related to CV events (p = 0.046) and prediabetes/diabetes were associated to TE events (p = 0.035). A tendency toward increased mortality in the patients with impaired glucose homeostasis at the last follow-up was also noted (p = 0.052) (Data not shown).

The entire CD cohort presented an increased mortality, with a SMR of 3.22 (95%CI [1.70; 5.60], p = 0.002). Mortality was significantly higher in the patients with persistent disease (SMR 4.99, 95%CI [2.15; 9.83], p < 0.001), but it was similar to that of the general population in the patients in remission (SMR 1.66, 95%CI [0.34; 4.85], p = 0.543). The finding was independent of the timing or the modality used to achieve cortisol control; for the early remission group the SMR was 2.15 (95%CI [0.36; 7.11], p = 0.477) and for the late remission group it was 1.14 (95%CI [< 0.01; 5.62], p = 1.0). The length of remission period was 82 [38–139] for the early remission group vs 85 [21–136] for the late remission one.

## Discussion

Study findings have confirmed that CD patients have a higher mortality and, as previously observed, the most common cause of death in these patients was, first of all, CV events and, secondly, infections [9]. Although there were no fatal TE events in our cohort, that type of complication was the most frequent one. As expected, the patients with persistent CD presented significantly increased mortality with respect to the general population. At the last follow-up examination the CD patients in remission had a mortality rate that was comparable to that of the general population regardless of the number of treatments needed to achieve remission. The finding is in contrast with the results of a multicentre study examining patients with more than 10 years of remission that reported finding a normal life expectancy only in the patients who achieved an early remission following a single TSS [12]. The better life expectancy in our series may be explained by an extensive use of cortisol-lowering medications in our centre during active phases of CD. There was moreover at least a partial control in the late remission group during over 70% of the years assessed; this might have had a positive effect on the overall survival rate (data not shown). Furthermore, our study considered relatively recent years when significant improvement in timely diagnosis and available medical therapies have been made [9]. Lastly, being monocentric, our study showed a homogenous management of comorbidities that by contrast, is in highly unlikely in a retrospective international study. Since cardiovascular and metabolic risk factors related to cortisol-excess are major determinant of mortality in CD, the latter point is of the outmost importance.

Survival was positively influenced in our cohort by a younger age at diagnosis, the presence of a microadenoma at baseline [9] and a remission status at the last follow-up examination. As expected, an elevated number of comorbidities increased mortality, and as has been previously reported, arterial hypertension, in particular, reduced survival [28]. A tendency toward increased mortality was also noted in connection to impaired glucose homeostasis, but data on this topic are still controversial [8, 10, 12, 28, 29].

Cortisol excess atherosclerotic risk leading to CV events are closely liked. Beyond cortisol’s direct action on the tissues, this association is probably related to a clustering of several metabolic complications such as insulin resistance, arterial hypertension, dyslipidaemia and overweight commonly present in CD patients [30, 31]. Indeed, the patients presenting multiple comorbidities, especially arterial hypertension and dyslipidaemia, showed more CV complications. CV events were also more frequent in the patients with persistent hypercortisolism, and, as observed in general population in the elderly and in the smokers [32].

Older age at the time of diagnosis and dis-glycemia at the last follow-up examination were found to be related to TE events. It was instead impossible to identify predictors of infective complications. Although most TE and IN events occurred during active disease, remission did not significantly reduce these complications. The finding is in line with the data of a recent study focusing on a Swedish population reporting that CD patients present a higher risk of sepsis and thromboembolism even during long term remission [33]. Moreover, it is worthy of note that most of the TE events (52.6%) were accompanied by a concomitant risk factor such as recent surgery. These data highlight the importance of adequate prophylaxis in CD patients facing prothrombotic conditions such as those linked to a perioperative period [3, 34]. Disease severity at the baseline did not affect the patients’ complications or survival; the finding is not entirely surprising as the degree of cortisol excess does not necessarily correlate with the severity of the clinical picture [2].

The patients who achieved remission in our cohort showed an overall improvement in all the cortisol-related comorbidities. Hypertension was the most prevalent complication at the time of diagnosis, while overweight, which persisted in approximately 50% of the cases after remission, became by far the most frequent comorbidity. Glucose homeostasis alterations were the least prevalent at the time of diagnosis, although an underestimation is probable, as only fasting glycaemia or glycosylated haemoglobin were evaluated in most cases and provocative testing for hypercortisolism was not carried out [35].

With regards to demographic features, for the most part our patients were diagnosed during their third/fourth decade of life and they were prevalently female, in line with previous reports [36]. Most cases were due to a pituitary microadenoma (80% of the cases in our patients), including non-visible lesions on the MRI.

As far as treatment was concerned, the remission rate after the first TSS was quite low with respect to what would be expected at a tertiary centre; the finding can be explained by the fact that many of the patients studied had been referred to our unit after undergoing unsuccessful pituitary surgery elsewhere. However, the assessment of surgical performance in various centres goes beyond the aim of the present study. As expected, a second TSS was less successful than the first one, but the rate of success found in our patients was in line with literature data [37]. Although the immediate remission rate after a second TSS was comparable to the long term outcome of radiotherapy, a quarter of the patients experienced a relapse just as they did after the first surgery [17]. Regarding the risk of developing hypopituitarism was concerned, no significant difference was found between the two approaches. These data have confirmed that both re-intervention and radiation treatment can be considered valid second-tier options, and a case by case approach should be adopted. Pre-operative medical treatment with cortisol-lowering medications did not improve the surgical outcomes, regardless of its effectiveness in controlling cortisol excess, in line with data by the European Registry on Cushing’s Syndrome (ERCUSYN) [38].

At the last follow-up examination, no differences in disease control were found when the treatment targets (pituitary vs adrenal) of the patients were compared. A higher control rate of hypercortisolism during active CD was found over time, possibly reflecting better drug dose titration and the widening landscape of available drugs with over two thirds of the patients presented completely controlled UFC at last examination. The fact that only one third of our patients achieved circadian rhythm restoration confirmed the previously reported difficulty in normalizing this parameter [39–41]. Interestingly, TE were more frequent when LNSC was uncontrolled and the same tendency was observed for survival, confirming the better outcome of patients with rhythm restoration [8]. Although only the last available value of LNSC was assessed, this finding might potentially turn the spotlight on the importance of LNSC normalization during medical treatment [42], but further studies are required to confirm these data.

In line with previous reports, more than one third of the patients who underwent BA developed CTP-BADX/NS [18]. Although BA seems to immediately control hypercortisolism, this benefit should be carefully weighed against the risk of permanent adrenal insufficiency and CTP-BADX/NS. The patients received minimal doses of glucocorticoid replacement treatments following BA to avoid both over- and under treatment that might negatively impact survival [43], and this might explain why BA was not associated to increased mortality as observed in other series [44]. Unilateral adrenalectomy was performed in selected cases when a large adrenal nodule, probably provoked by chronic ACTH stimulation [45], was found. Interestingly, two patients who had previously undergone radiation treatment of the pituitary achieved disease remission after this surgery. The “transition” from pituitary to adrenal hypercortisolism after long standing ACTH-stimulation on adrenal nodules in CD patients has already been described by other investigators, and it may explain our findings in the patients studied [46].

The study’s retrospective single-centre nature represents its primary limitation. Its other important limitation, the relatively low number of cases and deaths examined, is of course linked to the condition’s rarity. Being a monocentric study does, on the other hand, have its advantages as it ensures that the treatment strategies, comorbidities evaluation and management are homogeneous. Furthermore, data on comorbidities, disease activity, type of cortisol lowering medications and comorbidities are available for most of our cohort. Besides, a potential protective effect of tailored medical therapy to reduce cortisol levels seems to reduce some complications and, to a less extent, overall mortality, especially when circadian cortisol secretion is restored. Further studies are still required to confirmed these latter findings.

To conclude, active CD is characterized by increased morbidity and mortality, but disease remission seems to restore a normal life expectancy regardless of the timing and type of treatment used to achieve it. Thus, our aim as physicians is to pursue this goal by any means. Conversely, persistent cases seem to maintain an increase mortality, despite the use of effective cortisol lowering medications. Clearly persistent CD-related comorbidities require opportune monitoring and prompt management.

### Supplementary Information

Below is the link to the electronic supplementary material.

Supplementary file1 (DOCX 327 KB)

## Data Availability

Raw data are available from the corresponding author upon reasonable request.
