# Supplementary material for: Complications and mortality of Cushing’s disease: report on data collected over a 20-year period at a referral centre
Source: Pituitary. 2023 Jul 26;26(5):551–60. doi: 10.1007/s11102-023-01343-2 (PMC10539191; doi:10.1007/s11102-023-01343-2)

**Complications and mortality of Cushing’s disease: report on data collected over a 20-year period at a referral centre**

*Authors:* Alessandro Mondin^1^, Filippo Ceccato^1^, Giacomo Voltan^1^, Pierluigi Mazzeo^1^, Renzo Manara^2^, Luca Denaro^3^, Carla Scaroni^1^, Mattia Barbot^1^

^1^ Endocrinology Unit, Department of Medicine-DIMED, University Hospital of Padova, Padova, Italy

^2^ Neuroradiology Unit, University Hospital of Padova, Padova, Italy

^3^ Academic Neurosurgery, Department of Neurosciences, University of Padova, Padova Italy

*Short title:* Monocentric experience on treatment, complications and mortality of Cushing’s Disease

*Key words:* Cushing’s disease; mortality, morbidity, cardiovascular complications, survival

*Word count: main text 4386 words without abstract and references, abstract 275 words, 2 tables, 3 figures, 43 references.*

**Supplementary data**

**Material and methods**

The patient’s disease status was defined by the finding of the persistence or the remission of hypercortisolism at the time point considered. The patient was considered in remission if he/she had normal UFC and LNSC for at least 12 months after successful surgery, either pituitary or adrenal (bilateral adrenalectomy, BA), or in case of medical treatment discontinuation and normal hormonal values after radiotherapy. Post-surgical persistence was defined as the presence of hypercortisolism at any time within the 12-month period following the operation. Recurrence was defined as the finding that two out of the DST, UFC and LNSC test results were abnormal 12 months or more following successful pituitary surgery (17). Situations in which the patients achieved remission after the first TSS without any relapses were classified as “early remissions”. Patients not treated surgically or who presented persistent/relapsing disease after the first TSS that achieved remission via second-tier options (repeated surgery, radiotherapy and/or BA) were classified as “late remissions”.

In case of post-operative cortisol suggestive for remission, patients were tested at 1-3-6-12 months after pituitary surgery then yearly once pituitary-adrenal axis recovered or every 6 months for patients with increased risk of recurrence (e.g. those with persistent positive response to desmopressin test). In case of persistent disease, patients were evaluated every 2 to 6 months depending on comorbidities and disease control.

Disease activity during active CD was assessed on the basis of UFC levels. The patients were categorized as: controlled (UFC < ULN), partially controlled (UFC < 2xULN) or uncontrolled (UFC > 2 x ULN). Whenever possible, the effect of therapy on LNSC values was also evaluated.

The progression to Nelson’s Syndrome was evaluated on the basis of pituitary adenoma remnant growth (i.e., corticotroph tumor progression leading to Nelson’s syndrome after bilateral adrenalectomy, CTP-BADX/NS) (18). As these patients did not present relapsing hypercortisolism they were included in the remission group for statistical analysis, and no further treatments to control pituitary tumour growth were taken into consideration.

As far as comorbidities were concerned, the patients were considered hypertensive when systolic blood pressure ≥ 140 mmHg and/or diastolic blood pressure ≥ 90 mmHg, or if they were receiving ongoing antihypertensive treatment (19). Glucose homeostasis alterations were classified in accordance with the most recent American Diabetes Association standard of care (20). Type 2 diabetes mellitus (DM) was diagnosed after two separate findings of either: fasting plasma glucose ≥ 7 mmol/L, 2-h plasma glucose ≥ 11.1 mmol/L during 75g oral glucose tolerance test (OGTT), a glycated haemoglobin ≥ 48 mmol/mol or a random plasma glucose ≥ 11.1 mmol/L in a symptomatic patient. Prediabetes was diagnosed either as impaired plasma glucose (IFG, fasting plasma glucose between 5.6 and 6.9 mmol/L) or impaired glucose tolerance (IGT, 2-h plasma glucose during 75g OGTT between 7.8 and 11.0 mmol/L). In accordance with the body mass index (BMI), the patients were classified as being normal (18 – 25 kg/m2), overweight (≥ 25 kg/m2) or obese (≥ 30 kg/m2) (21). Dyslipidaemia was diagnosed according to the National Program Adult Treatment Panel III (NCEP ATP III) criteria (22) or according to the European Society of Cardiology (ESC) guidelines for patients with calculated low-density lipoproteins (LDL) above the recommended target for their CV risk (23) or if the patient was receiving ongoing lipid-lowering treatment. Hypopituitarism was diagnosed according to the Endocrine Society guidelines (24).

**Standardized mortality ratio calculation**

Standardized mortality ratio (SMR) was calculated based on indirect age standardization in order to compare the observed deaths in our CD population with the expected number of deaths in the general population, as previously described by Breslow and coll (26).

We obtained age-specific yearly death rates in the Italian population from 2011 to 2020 from the database of Italian National Institute of Statistics (age-specific data about previous years could not be retrieved). Expected deaths were computed for every year and 5-year age class as follows:

$$E_{ij}=d_{ij}*n_{ij}$$

Where *E* is the expected number of deaths for the *i* -th year and *j* -th age class, *d* is the death rate for the *i* -th year and *j* -th age class and *n* is the number of subjects in our population for the *i* -th year and *j* -th age class.

Then the SMR was calculated as follows:

$$SMR=\frac{O}{\sum E_{ij}}$$

Where *O* is the observed number of deaths in the study population during the years considered.

We conducted a Fisher exact test to assess significant difference from general population and calculating the 95% confidence interval (95% CI) for SMR. We used an open-source calculator to perform Fisher exact test (http://www.openepi.com).

***Supplementary table 1.*** *Distribution of medical treatments at last follow-up. A: adrenal target; P: pituitary target; M: mixed target, both adrenal and pituitary; n: number of patients. * Among these three patients: a patient was on a combination of ketoconazole, metyrapone, cabergoline and mitotane and another patient was receiving metyrapone, cabergoline and mitotane; for the third patient, harbouring an aggressive pituitary adenoma, a combination treatment of temozolomide, pasireotide and metyrapone was carried out.*

| **Monotherapy** | | | **Combination treatment** | | |
| --- | --- | --- | --- | --- | --- |
| **Drug** | **Target** | **n** | **Drugs** | **Target** | **n** |
| Metyrapone | A | 10 | Ketoconazole + Metyrapone | A | 6 |
| Ketoconazole | A | 6 | Ketoconazole + Cabergoline | M | 4 |
| Osilodrostat | A | 3 | Metyrapone + Cabergoline | M | 3 |
| Pasireotide | P | 7 | Metyrapone + Pasireotide | M | 3 |
| Cabergoline | P | 2 | Other* | M | 3 |

***Supplementary table 2.*** *Prevalence of comorbidities at diagnosis in patients achieving remission and in those with persistent disease at last follow-up.*

|  | **Remission** | **Persistence** | **p** |
| --- | --- | --- | --- |
| **Baseline hypertension** | 50/63 (79.4%) | 38/42 (90.5%) | 0,22 |
| **Baseline dyslipidaemia** | 23/46 (50.0%) | 13/20 (65.0%) | 0,38 |
| **Baseline diabetes/prediabetes** | 25/55 (45.5%) | 17/28 (60.7%) | 0,07 |
| **Baseline overweight** | 38/54 (70.4%) | 23/28 (82.1%) | 0,17 |

***Supplementary table 3.*** *Disease activity in patients presenting persistence at the last follow-up and in patients achieving a late remission. n = number of patients.*

|  | **n** | **Total of the assessed years** | **Completely controlled** | **Partially controlled** | **Not controlled** |
| --- | --- | --- | --- | --- | --- |
| **Persistence** | 48 | 463 | 39.1% | 19.7% | 41.3% |
| **Late remission** | 33 | 170 | 42.4% | 31.2% | 26.5% |

***Supplementary table 4.*** *Percentages of hormone deficiencies and their clustering at the last available follow-up, including post-BA hypocortisolism. n: number of patients; tot: total of patients considered.*

| **Deficient axis** | **n/tot (%)** | **Deficit clustering** | **n/tot (%)** |
| --- | --- | --- | --- |
| Corticotroph | 23/124 (20.2%) | Four | 1/126 (0.8%) |
| Somatotroph | 21/118 (17.7%) | Three | 5/126 (4.0%) |
| Thyrotroph | 10/115 (8.7%) | Two | 9/126 (7.1%) |
| Gonadotroph | 9/117 (7.7%) | One | 27/126 (21.4%) |
|  |  | None | 84/126 (66.7%) |

***Supplementary figure 1.*** *Remission and relapse rates of pituitary surgeries in our population. n: number of patients undergoing surgery.*


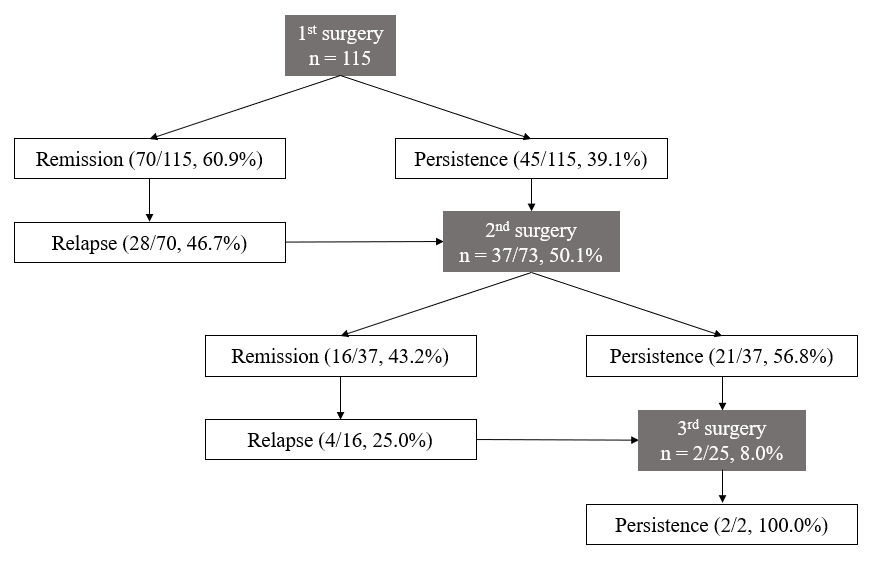


***Supplementary figure 2.*** *Surgical outcome (remission/persistence) based on biochemical control during medical treatment prior to 1^st^ (A) and 2^nd^ (B) surgery.*


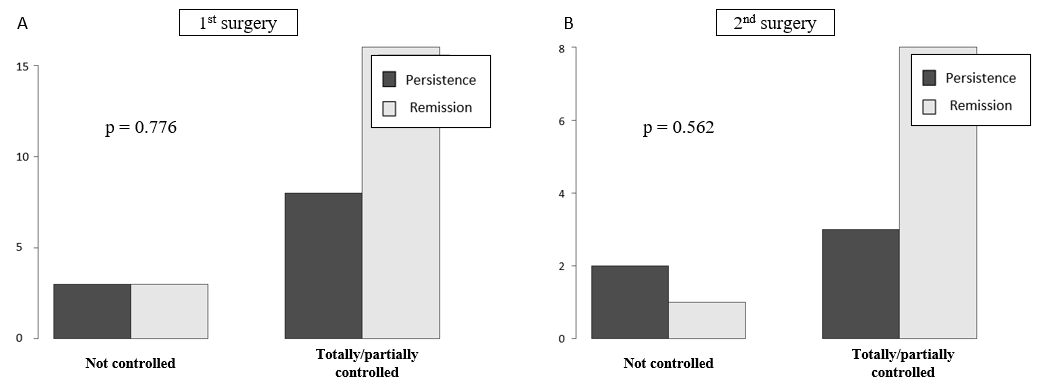


***Supplementary figure 3.*** *A) Pituitary deficits in patients who received second surgery and patients who did not; in order to reduce the risk of bias all patients considered underwent a prior pituitary surgery and did not receive any pituitary irradiation. B) Pituitary deficits in patients who received radiotherapy and those who did not; in order to reduce risk of bias we included only patients who also received at least one previous pituitary surgery. In both comparisons (A and B) patients presenting isolated glucocorticoid deficiency after BA were excluded. PD: at least one pituitary deficit.*


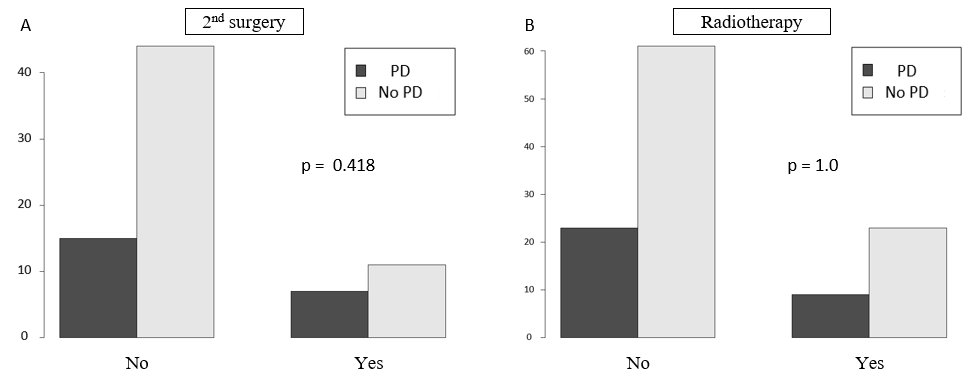


***Supplementary figure 4.*** *Kaplan Meier curves for A) thromboembolic events and B) survival, based on circadian rhythm restoration at the last follow up. FU: follow-up; TE: thromboembolic events. * p<0.05*


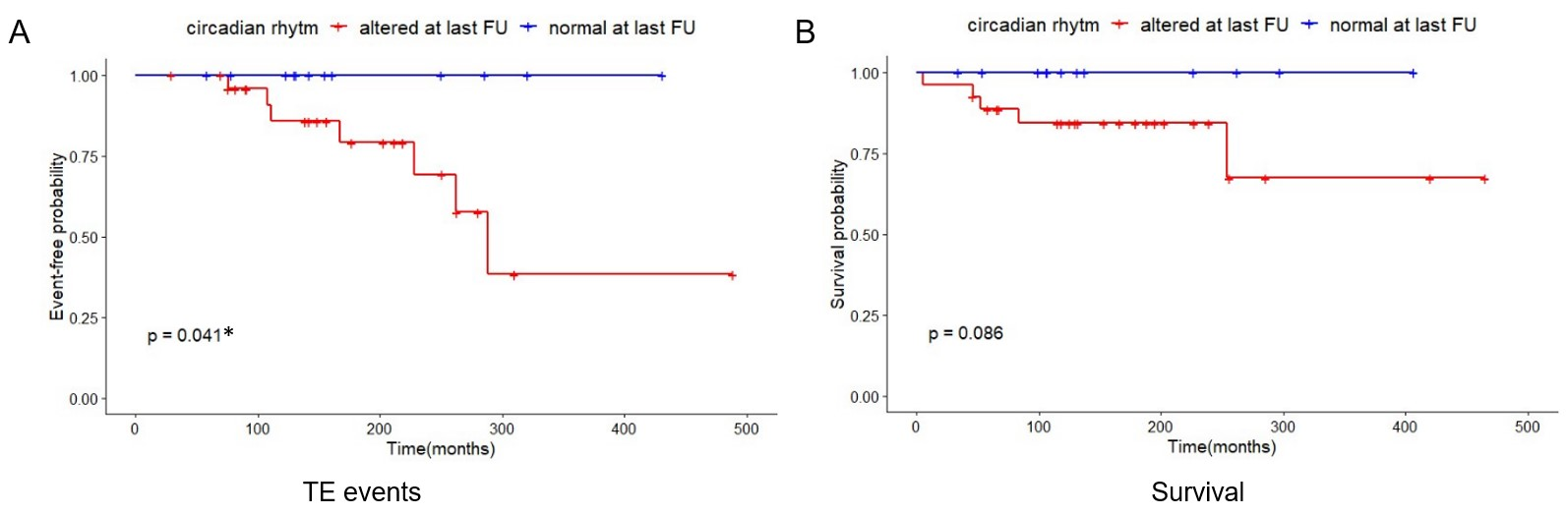

Supplement: Supplementary file 1 — Supplementary file1 (DOCX 327 KB) [file 11102_2023_1343_MOESM1_ESM.docx]
